# Optimal Timing Regularly Outperforms Higher Coverage in Preventative Measles Supplementary Immunization Campaigns

**DOI:** 10.3390/vaccines12070820

**Published:** 2024-07-22

**Authors:** Katherine A. Rosenfeld, Kurt Frey, Kevin A. McCarthy

**Affiliations:** Institute for Disease Modeling, Bill and Melinda Gates Foundation, Seattle, WA 98109, USA

**Keywords:** measles, immunization, modeling

## Abstract

Measles threatens the lives and livelihoods of tens of millions of children and there are countries where routine immunization systems miss enough individuals to create the risk of large outbreaks. To help address this threat, measles supplementary immunization activities are time-limited, coordinated campaigns to immunize en masse a target population. Timing campaigns to be concurrent with building outbreak risk is an important consideration, but current programmatic standards focus on campaigns achieving a high coverage of at least 95%. We show that there is a dramatic trade-off between campaign timeliness and coverage. Optimal timing at coverages as low as 50% for areas with weak routine immunization systems is shown to outperform the current standard, which is delayed by as little as 6 months. Measured coverage alone is revealed as a potentially misleading performance metric.

## 1. Introduction

Since the 1900s, measles has killed tens of millions. Today, the threat of measles varies widely between low-, middle-, and high-income countries, and in the most challenged regions the disease threatens a similar order of children, mostly under the age of 5 and often the most vulnerable [1]. Measles supplementary immunization activities (SIAs) are an important programmatic tool to stop large outbreaks and interrupt transmission [2]. These are time-limited, coordinated campaigns to immunize en masse a target population that has been missed by the routine immunization (RI) system. SIAs can be run at the national or subnational scale, and fill immunity gaps by immunizing children with a dose of measles-containing vaccine (MCV) [3].

The timeliness of these campaigns has been known to be a critical component of campaign planning and execution [4,5,6,7], particularly due to the high infectivity of the measles virus and the rapidly escalating nature of outbreaks (e.g., [8]). The true impact of a measles campaign is the burden averted by preventing/precluding outbreaks. However, the burden averted cannot be directly measured and must be estimated from modeled counterfactuals. Currently, the standard proxy for measuring a campaign’s quality is 95% coverage both overall and in every district [9]. While coverage is a critical measure of campaign quality, it only captures one side of the task: what proportion of children received an MCV dose through the SIA. However, it cannot capture whether the SIA reached them prior to infection by the circulating virus.

While the role of timeliness is generally appreciated, the campaign planning and financing cycle is long, and the actual date of campaign execution can be delayed from the epidemiologically optimal timing for many reasons, including civil conflict/insecurity, vaccine and device shortages, and cold-chain space [9]. This paper investigates the impact of delays and trade-offs between a campaign’s coverage and its timeliness.

SIAs are generally run in countries with endemic measles transmission after one birth cohort of susceptibles has accumulated [9]. This is often assessed as a balance between births, RI, and previous immunization campaigns [10,11]. Additionally, the impact of disease-induced immunity can be incorporated via epidemiological modeling [12]. Broadly speaking, these campaigns are run at a cadence of 2 to 5 years, depending on RI rates [13]. Campaigns are usually targeted to children in age cohorts likely to be susceptible because they have not been previously vaccinated via RI or SIAs or been infected with measles.

**Figure 1:**
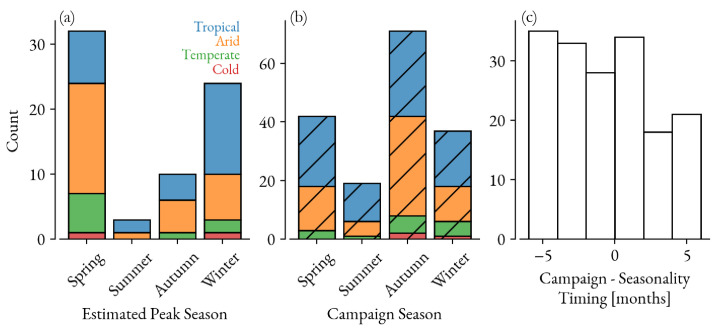
(**a**) Estimated measles peak seasons at the national level and further aggregated by Köppen–Geiger climate classification [21]. (**b**) The aggregated timing of SIA campaigns from 2000 to 2020. (**c**) The resulting offset distribution between campaign timing and the estimated seasonality.

It is possible to find and study examples of delayed SIA campaigns for specific countries (e.g., [14,15]), but with no comprehensive database it is instructive to consider the relative timing of campaigns and periods of high measles transmission when outbreaks are most likely to occur. Measles seasonality is well studied [6,16,17,18,19,20] and historical case data reveal a propensity for outbreaks in the spring and winter months. Figure 1 shows these peak seasons for measles transmission estimated from the World Health Organization’s (WHO) monthly measles data from 2000 to 2020 for 174 countries (see Section 2.1 for more details). However, historical SIA data from the WHO’s SIA calendar for the same time period reveal a more even distribution across the seasons of national campaign timings. The two distributions are significantly different from what one would expect based on epidemiological timing (KS test, $p=1.1\times 10^{-6}$). Furthermore, the resulting distribution of offsets from campaign timing to the estimated peak seasonality shows that there are many campaigns run during the peak season as well as a few months after.

Epidemiology is only one important component of deciding when to schedule campaigns along with a number of other considerations including resource availability, weather, and community factors [9]. The timeline for planning these campaigns is complex and involved; the WHO field guide details over 85 pages, with the steps involved including macroplanning, budgeting, microplanning, training, social mobilization, and monitoring [9]. Potential delays in any of these steps could propagate to a delay in the overall execution of the campaign. Although the highest quality campaign is always desired, there is a trade-off between a well-timed but potentially lower quality campaign and a higher quality campaign that is mistimed. This analysis provides quantitative assessments looking at near-term measles incidence as well as longer term patterns.

## 2. Materials and Methods

### 2.1. Seasonality Estimation

The monthly seasonality of measles transmission is roughly estimated for countries represented in the WHO’s monthly measles cases data by looking at the phase offset for the yearly periodic signal in the case data’s Fourier transform from 2000 to 2010; it is selected for countries who have had at least 100 cases over the time series. The monthly estimates are aggregated to seasons as reproduced in Figure 1. The results are consistent whether seasonality is estimated from the Fourier transform or the maximum monthly cases aggregated over the 20-year time series. The seasonality and campaign timing are compared by offsetting the campaign by 4 months and then performing a KS test.

### 2.2. Transmission Model and Building the SIA Calendar

Measles transmission dynamics were simulated using the Generic branch of EMOD ([22], v2.21). Simulations of a well-mixed population with varying RI were given the the model initialization and inputs described at the end of this section. This software has been calibrated and applied for country-specific models including measles [23], polio [24], malaria [25], and rubella [26]. For this analysis, a generalized model not calibrated to a single country but varied across parameters relevant for a broad range of locations was chosen. The model produces dynamic outbreak behavior where the periodicity and severity of outbreaks can maintain long-term stability or fluctuate over time. These dramatic changes in the transmission patterns are consistent with observations of measles and our mathematical understanding [18,27]. However, this causes a challenge for incorporating SIAs since the simulation parameters do not consistently determine the outbreak periodicity and behavior.

To build the SIA calendar, each simulation is initially run for 15 years. In post-processing, the first outbreak to occur after a 5 year period is identified as when the number of current infections exceeds the 95th percentile of active infections that occur over the entire simulation run. This metric is used throughout to identify the start of outbreaks in the simulation. A following round of simulations are launched that follow the initial transmission trajectory but with a rapidly implemented (1-day duration) SIA [9] scheduled 4 months before the start of the outbreak. This process can then be repeated to generate a calendar of effective SIAs.

SIA doses are age-targeted to children between 9 months and 5 years. Doses are first allocated to children who have received a dose via RI, reflecting an underlying assumption that children already reached by RI are also most accessible to outreach activities. This is an assumption that produces conservative bounds on the impact of SIA delays. Surveys suggest that the performance of SIAs in reaching children who are not immunized through the RI system varies between settings [28].

For the analysis, two values of RI coverage (30% and 70%) are considered to highlight the differences between low- and higher-coverage settings [29]. SIA coverage values are run at 50%, 70%, and 95%. The highest SIA coverage reflects the current recommendation of 95% coverage both on aggregate and in each district [9], which is more stringent than measles elimination strategies that aim for 95% coverage in combination between RI and SIAs [30,31].

The setting is initialized with 1 million individuals with an age distribution equilibrated for a given crude birth rate and the age-based mortality of Nigeria. A reproductive number, $R_{0}$, of 14 is assumed, consistent with a exponential infectivity distribution with daily rate equal to 14/8 and a Gaussian infectious period with mean of 8 days and standard deviation of 2 days. The latent period is assumed to be normally distributed with mean of 10 days and standard deviation of 2 days. Maternal protection was incorporated with initial susceptibility following the sigmoid $1/\left(1+\exp\left((50-\mathrm{age}\;\mathrm{in}\;\mathrm{days}\right)/50\right)$.

The model includes a constant rate of imported infections, and daily values of 0.03 and 0.1 are considered for a sensitivity analysis. The results are shown for a value of 0.1, but qualitative results do not change with the lower importation rate. Crude birth rates were chosen randomly from a range of 25 to 40 per 1 thousand people in the population. Transmission seasonality was incorporated as a sinusoidal forcing factor on infectivity, with amplitude varying between 20% and 100%. Note that for the upper bound of 100%, infectivity approaches zero for a small time during the year. Analyses performed with an amplitude ranges of 0% to 50% had the same qualitative results. There were 256 simulations run over this space for each scenario analyzed.

SIA dose allocation was carried out such that children reached by RI would receive an SIA dose before a child who did not receive an RI dose. There is no MCV2 dose incorporated into the simulation. At MCV1 rates of 30% and 70%, and given an MCV1 seroconversion probability of 85% at 8 months [32], susceptibility is dominated by zero-dose children, not MCV1 recipients who failed to seroconvert. MCV2 is thus not expected to qualitatively change the results presented here.

## 3. Results

**Figure 2:**
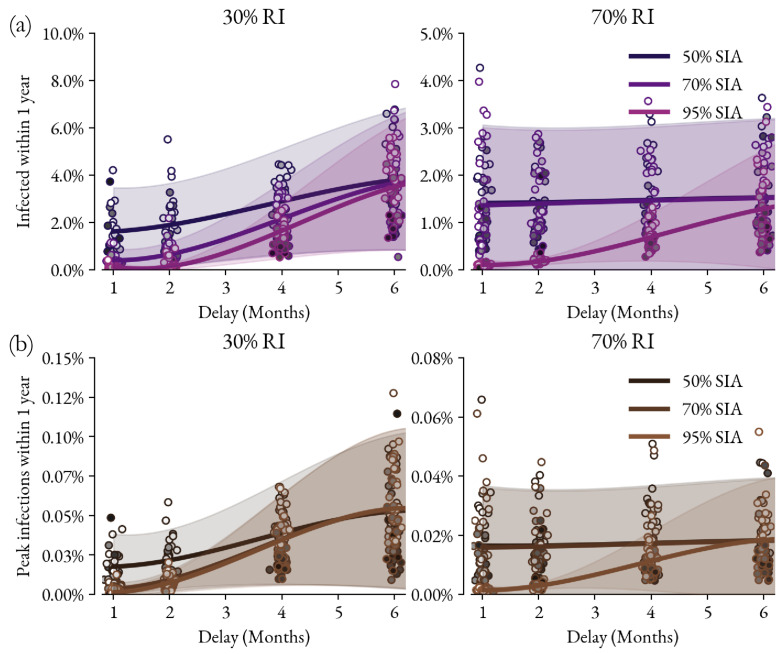
Resulting outbreak size (number of infections for a year following when the SIA was planned) and intensity (peak number of infections for that same time period) are measured in both total infections (**a**) and peak number of infections (**b**). Each circle represents a simulation (N = 256) with the circle’s shading indicating the birth rate (scaling from 25 to 40 per 1 k people per year). Curves are Gaussian process regression fits to the simulation results and shaded regions are 95% CIs from the fit; 70% and 95% lines are indistinguishable for a 70% RI coverage scenario.

Measles transmission dynamics were simulated in a well-mixed population with routine immunization, vital dynamics, and an optimized calendar of SIAs. The trade-off between SIA coverage and timeliness in Figure 2 is assessed by two different metrics. The top two panels show the outbreak size as measured by the total number of people infected over the next year after the SIA was scheduled to occur. The bottom two panels show the outbreak intensity as measured by the maximum peak daily incidence over that year. These metrics are calculated for each simulation as the SIA is delayed by 1, 2, 4, and 6 months. The results for 50%, 70%, and 95% coverage SIAs are visualized with added noise in the x-dimension (time) for the visualization. The mean trend over the entire set of simulations is depicted by the solid line; shaded regions correspond to the 95% confidence intervals. For the 30% RI coverage scenario, the 50% SIA delayed by 1 month has a smaller and less intense outbreak than the 95% SIA delayed by 6 months. This trade-off only reverses after 5 months of delay for the 50% SIA. For the 70% RI coverage scenario, the 50% and 70% SIAs do not change appreciably with the delay due to our modeling assumption that SIA doses are administered first to children who have received a dose via RI (see Section 2.2).

**Figure 3:**
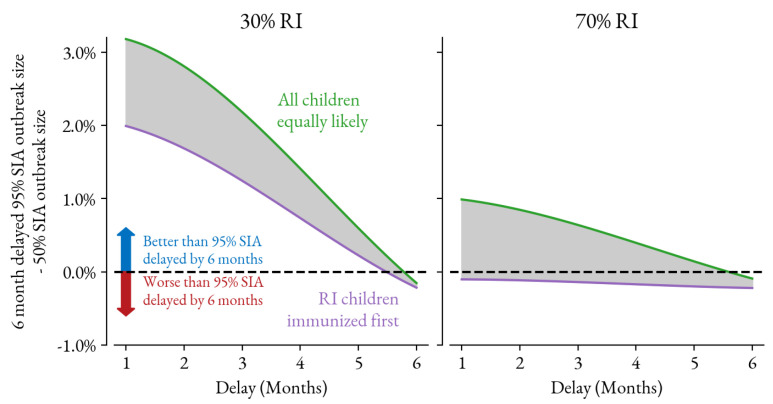
Different dose associations for previous immunization will impact how much, if at all, the well-timed but lower-coverage SIA will perform compared to the delayed, higher-coverage SIA. In these figures, the difference in outbreak size between the 95% coverage SIA delayed by 6 months and the 50% SIA delayed between 1 and 6 months is plotted for two bounding assumptions. The purple line shows the results for SIA doses going first to children who received a dose via RI. The green line shows results for all children being equally likely to receive a dose during the SIA.

The importance of the dose association assumption is explored in Figure 3. The absolute difference in outbreak size (calculated as percentage of the population) is shown between the 95% SIA delayed by 6 months and the 50% SIA as it is delayed by 1, 2, 4, and 6 months. However, the mean trend is compared across the simulation suite for two bounding assumptions: (1) SIA doses first go to children who have received a dose via RI and (2) all children are equally likely to receive an SIA dose. This calculation shows how the lower-coverage, timely SIA will outperform the higher-coverage, delayed SIA in higher RI coverage settings if the campaign is able to effectively reach zero-dose children, specifically children who have not received a measles vaccine.

**Figure 4:**
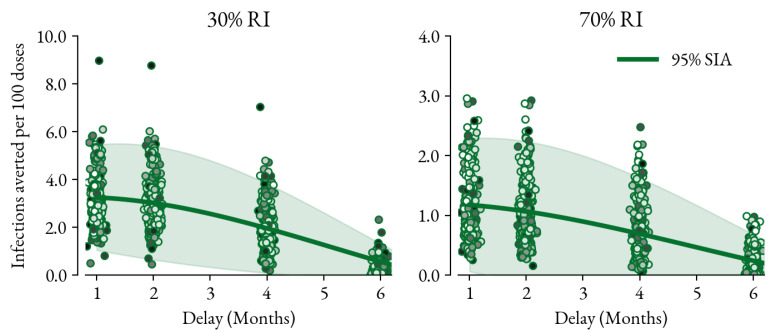
Dosage impact of the 95% coverage SIA as measured by infections averted per 100 campaign doses as it is delayed from 1 to 6 months. The infections were counted over the year following the planned SIA compared to if no SIA was executed. The impact per dose of the SIA is reduced by a factor of almost 3 when moving from a 1- to 6-month delay.

The last metric considered is how the cost-effectiveness of the high-coverage SIA is affected by delay. Figure 4 shows the infections averted per 100 campaign doses as the 95% SIA is delayed by 1, 2, 4, and 6 months. The cost-effectiveness of the 95% SIA is reduced by almost a factor of 3 as it is delayed by 1 to 6 months. This is due to the fact that the campaign doses arrive too late to avoid the outbreak and are administered to children who have been infected and therefore acquired natural immunity.

**Figure 5:**
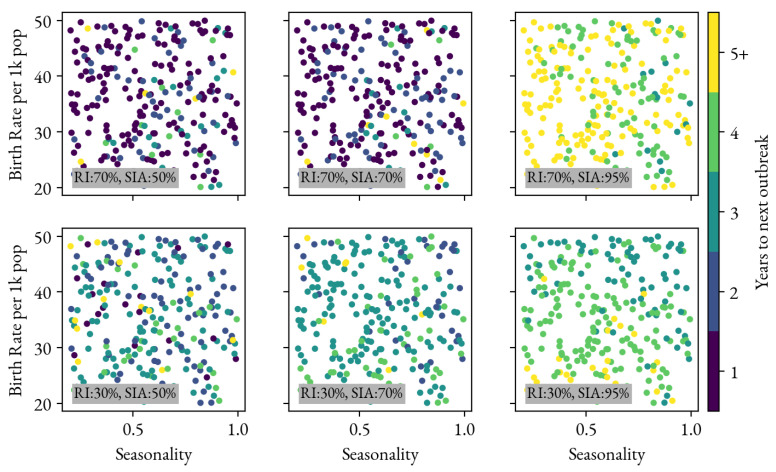
Period in years between the SIA and the next outbreak depicted across the range of simulations considered in this analysis.

Along with the immediate effect of the SIA in reducing the next year’s infections, longer-term effects are considered by showing the number of years until the next outbreak in Figure 5. Each individual sub-panel shows results for RI and SIA coverage level combinations. Each simulation is plotted as an individual circle whose color indicates the number of years until the next outbreak. These SIAs are not delayed. As seen in Figure 2, an SIA coverage of less than 70% has limited effect for the higher RI coverage scenario (70%). This produces the counter-intuitive result that simulations with 30% RI and 70% SIA coverage have more time between outbreaks than simulations with 70% RI and (essentially useless due the dose association assumption) 50% or 70% SIAs. However, looking across the different coverage scenarios, higher-coverage and timely SIAs will not only reduce burden for the coming year (Figure 2) but also increase the number of years until the next outbreak. A dependence on birth rate can also be observed. Both of these results are consistent with previous studies (e.g., [13]).

## 4. Discussion

SIAs are a proven and trusted tool for measles outbreak prevention where sub-optimal RI leaves immunity gaps. However, a tool can only be as good as its application. In the case of endemic measles transmission, it must be applied in a timely manner. This analysis demonstrates how SIAs performed on time outperform delayed SIAs even if the timely SIA has significantly lower coverage. The cost-effectiveness of the high-coverage SIA is also greatly reduced by delay. These results suggest that coverage is not necessarily the only metric that should be considered to evaluate SIA performance and impact. Furthermore, coverage should be considered within the larger context of transmission patterns to duly evaluate how effective the SIA is at reducing susceptibility. This becomes increasingly important given the recent backslide in observed RI systems and surveillance [33]. However, it is important to keep in mind that a substantial reduction in measles mortality over the past decade is well documented, although rarely incorporating epi-linked case and RI histories (e.g., [34]). Vaccination, through RI systems and SIAs, plays an important role, but other interventions (targeting, e.g., improved women’s health, management of acute malnutrition, and early breastfeeding) can also have a significant impact on reducing measles mortality and morbidity [34].

There are several limitations to this analysis. First, the role of MCV2 is not considered for clarity of the discussion and interpretation. Second, clustering or transmission correlation between susceptible groups that might arise from low-coverage settings (e.g., geographic, communal) is not included. This could be investigated via a setting-specific model calibrated to observed transmission patterns. The choice to use a more generic model to investigate general trends and patterns sacrificed the ability to capture this issue. Heterogeneity in coverage, whether it is derived from remoteness [35], conflict [36], or intersectional factors [37], would also impact the corresponding impact of the SIA. Finally, the simulation setup was designed such that there was complete certainty about the imminence of a measles outbreak, and thereby maximizes the observed effects of delay. In practice, campaigns must be planned over long horizons; measles outbreaks often fall into periodic patterns [38,39] and the 1-birth-cohort-unvaccinated rule of thumb provides a good proxy for susceptibility sufficient to support an outbreak, since measles outbreaks are not perfectly predictable. However, timeliness remains a necessary component for an effective SIA.

Reaching zero-dose and under-immunized children is an important goal and necessary component of measles outbreak prevention and control. Not only is this an equitable aim, it is central to reducing measles morbidity and mortality world-wide. As part of the IA2030 strategy, the prioritization of zero-dose children for SIA campaigns remains critical [40]. However, in the case of measles SIAs in outbreak-prone settings, this analysis strongly suggests that coverage and timeliness are intricately intertwined. Higher-coverage SIAs will naturally outperform lower-coverage ones in a strict comparison. However, if epidemiological analyses indicate that outbreaks may be imminent and the necessary planning steps to achieve higher coverage would incur significant delays, it may be better to prioritize timeliness for coverage.

## Data Availability

Scripts and simulation outputs will be available on zenodo: https://doi.org/10.5281/zenodo.7764622. Source code for EMOD is available on GitHub: https://github.com/InstituteforDiseaseModeling/EMOD-Generic (accessed on 1 July 2024).

## Abbreviations

The following abbreviations are used in this manuscript:

EMOD	Epidemiological MODeling software
MCV	Measles-Containing Vaccine
RI	Routine Immunization
SIA	Supplementary Immunization Activity
WHO	World Health Organization
